# Feasibility of radical cardiac‐sparing, treatment planning strategies for patients with locally advanced, non‐small cell lung cancer

**DOI:** 10.1002/acm2.13784

**Published:** 2022-10-13

**Authors:** Joshua P. Kim, Jake Dewalt, Aharon Feldman, Khaled Adil, Benjamin Movsas, Indrin J. Chetty

**Affiliations:** ^1^ Department of Radiation Oncology Henry Ford Health System Detroit Michigan USA

**Keywords:** cardiac, NSCLC, treatment planning

## Abstract

**Purpose:**

A set of treatment planning strategies were designed and retrospectively implemented for locally advanced, non‐small cell lung cancer (NSCLC) patients in order to minimize cardiac dose without compromising target coverage goals.

**Methods:**

Retrospective analysis was performed for 20 NSCLC patients prescribed to 60–66 Gy that received a mean heart dose (MHD) ≥10 Gy. Three planning approaches were designed and implemented. The first was a multi‐isocentric (MI) volume‐modulated arc therapy (VMAT) approach (HEART_MI) with one isocenter located within the tumor and the second chosen up to 10 cm away longitudinally. The second was a noncoplanar (NCP) VMAT approach (HEART_NCP) utilizing up to three large couch angles and a standard arc at couch 0. The final planning strategy took a mixed approach (HEART_HYBRID) utilizing the HEART_NCP strategy for two thirds of the treatment combined with a plan utilizing a pair of opposite‐opposed gantry angles for the remaining treatments. Investigational plans were compared to original plans using dose–volume histogram metrics such as organ volume receiving greater than *x* Gy (*Vx*) or mean dose (*D*mean).

**Results:**

Although there was a small but statistically significant decrease in internal target volume coverage for HEART_MI plans and, conversely, a statistically significant increase for HEART_NCP plans, all generated plans met physician‐prescribed target constraints. For heart dose, there were statistically significant decreases in all heart metrics and particularly MHD for the HEART_MI (9.8 vs. 15.4 Gy [*p* < 0.001], respectively), HEART_NCP (9.2 vs. 15.4 Gy [*p* < 0.001]), respectively), and HEART_HYBRID (7.9 vs. 15.4 Gy [*p* < 0.001], respectively) strategies.

**Conclusions:**

The strategy providing the best compromise between plan quality and cardiac dose reduction was HEART_NCP, which produced MHD reductions of 37.6% ± 12.9% (6.2 ± 3.4 Gy) relative to original plans. This strategy could potentially reduce adverse cardiac events, leading to improved quality of life for these patients.

## 1 INTRODUCTION

Lung cancer is the leading cause of cancer death for both men and women in the United States, and the most common type of lung cancer is non‐small cell lung cancer (NSCLC), which comprises 80%–85% of all lung cancer cases.^1^ Traditionally, radiotherapy with concurrent chemotherapy has played a critical role in the treatment of patients with inoperable, locally advanced NSCLC due in large part to the extent of lesion, age of the patient, and other patient comorbidities.^2^ However, the outlook for patients undergoing locally advanced NSCLC treatments is poor with 5‐year overall survival rates hovering between 15% and ∼30%^3^, ^4^ and little data available about long‐term survival rates. There have been several avenues investigated to improve the outcomes for this population of patients with the most significant efforts related to dose escalation. The Radiation Therapy Oncology Group (RTOG) 0617 trial was designed in order to determine whether a 74‐Gy dose regimen would provide better survival outcomes than the traditional 60‐Gy dose regimen as well as to evaluate the effectiveness when cetuximab was added to concurrent chemotherapy.^5^ It was found that the patients in the 74‐Gy arm showed significantly worse overall survival outcomes relative to patients in the 60‐Gy arm (20.3 vs. 28.7 months).

One of the factors identified in multivariate analysis that could best explain the lower overall survival results in the high dose group was cardiac dose, particularly Heart *V*5 (volume of the heart receiving ≥5 Gy) and *V*30.^3^ Although the negative effects of high cardiac dose are well understood and studied in other types of radiotherapy such as in breast cancer,^6^, ^7^, ^8^ esophageal cancer,^9^ and Hodgkins lymphoma,^10^, ^11^ it has not been well studied within the context of locally advanced, NSCLC patients. This has historically been related to the poor prognosis and short typical lifespans after radiotherapy for this population of patients, which has led to the focus on factors that present more acutely and have been shown to have more of an immediate effect on patient outcomes such as esophageal and lung dose that could lead to esophagitis or radiation‐induced pneumonitis.^12^ Therefore, cardiac dose has not been as highly prioritized. For example, in the parameters for RTOG 0617, cardiac dose goals were quite general in only recommending the use of constraints such as <40 Gy to 100% of the heart.^5^

In the wake of the results from RTOG 0617, several groups have investigated whether the link between cardiac dose and overall survival could be replicated in independent groups. Although some studies,^13^ such as the Phase III ESPATUE trial that considered the effect of neoadjuvant chemotherapy followed by either surgery or definitive radiotherapy,^14^ were not able to verify the link between cardiac dose and overall survival, a series of retrospective single institution studies^15^, ^16^, ^17^, ^18^, ^19^ and meta‐analysis^20^ were able to support the link between cardiac dose and the incidence of cardiac adverse events. A retrospective study out of the University of Michigan^19^ found that on multivariate analysis, the presences of preexisting cardiac disease and mean heart dose (MHD) were found to be significantly related to the incidence of Common Terminology Criteria for Adverse Events^21^ grade 3 or higher cardiac adverse events. However, the incidence of disease progression (*n* = 71) exceeded that of cardiac events. Therefore, the need for cardiac sparing was illustrated, while also underscoring that this cannot be done at the expense of sacrificing target coverage. In another retrospective study out of the University of North Carolina,^17^ they found that MHD was significantly associated with the incidence of treatment‐related future cardiac events, which had reached greater than 10% of patients at 2 years. Likewise, in a study out of the Dana‐Farber Cancer Institute and Brigham and Women's Hospital,^15^ MHD (>10 Gy compared to ≤10 Gy) was significantly associated with an increased risk of major adverse cardiac events and all‐cause mortality for a subset of patients. This connection between the increase of symptomatic cardiac events and lower overall survival was investigated in a Rutgers study^18^ that found after multivariate analysis that MHD and baseline cardiac status were associated with an increased risk of symptomatic cardiac events and that the incidences of symptomatic cardiac events were independently associated with overall survival.

As the prognosis for locally advanced NSCLC patients has improved with the introduction of new treatment technologies such as intensity‐modulated radiotherapy, immunotherapy, and other targeted therapies,^22^ the results of these trials have lent credence to the argument that it is increasingly important to consider the effect of cardiac dose for these patients, while also maintaining a strong emphasis on neither sacrificing target coverage nor significantly increasing lung doses. At minimum, this seems to be especially true within the context of patients who have baseline cardiac disease prior to radiotherapy. There is also strong evidence from secondary analysis of the RTOG 0617 trial that there exists a correlation between cardiac dose–volume histogram (DVH) metrics and quality of life (QOL) metrics after 1 year.^23^ There exists then a need to utilize strategies for minimizing heart dose to a level that mitigates the risk of cardiac adverse events. Therefore, we implemented a set of three planning templates that could be used for this patient population to determine their effectiveness at reducing cardiac dose relative to conventional planning techniques for challenging cases. In this retrospective study, we evaluated the efficacy of these treatment planning strategies at minimizing dose to the heart without having to compromise target coverage. Each of these planning strategies is compared for their effectiveness at reducing cardiac dose and maintaining traditional target and organ‐at‐risk (OAR) (particularly lung) dose levels relative to the original treatment plans.

## 2 METHODS

### 2.1 Patients

Retrospective analysis was performed for 20 locally advanced, NSCLC patients (T3–T4, N0–N3). Using the MHD threshold that correlated with an increased level of major adverse cardiac events from the study by Atkins et al.,^15^ patients were selected who had originally received MHD > 10 Gy in the original plans that were used for the patients’ actual course of treatment. The patient population consisted of 7 men and 13 women with a median age of 70 years (range: 59–81) with either adenocarcinoma (*n* = 11) or squamous cell carcinoma (*n* = 9). Treatment site ranged from 10 left‐sided (6 lower lobe, 4 upper lobe), 8 right‐sided (6 lower lobe, 1 upper lobe, and 1 middle lobe), and 2 mediastinal lesions. All patients underwent radiotherapy using volume‐modulated arc therapy (VMAT) to a total dose of 60–66 Gy in 2 Gy per fraction (30–33 fx).

### 2.2 CT acquisition

All patients underwent 4DCT simulation using Brilliance Big Bore (Philips Health Care, Cleveland, OH) CT scanners to acquire phase‐based 4DCT thorax images with 10 phases reconstructed. The following parameters were used for scan acquisition: 120 kVp, 800 mA s, 512 × 512 in‐plane image dimensions, 1.17 × 1.17 mm^2^ in‐plane resolution, and 3‐mm slice thickness. The phase images were used to derive an average image that was used for treatment planning and a maximum intensity projection image to aid in contouring. A motion encompassing method was employed for target volume generation using an internal target volume (ITV)‐based approach. Based on individual patient characteristics, the expansion from the ITV to the PTV ranged from an isotropic expansion of 0.5 cm on the low end to asymmetric expansions of 0.5 cm axially and 1–1.5 cm along the cranial–caudal axis.

### 2.3 Treatment planning

**FIGURE 1:**
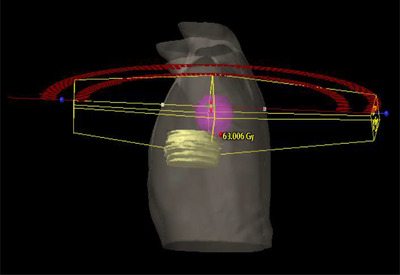
Typical treatment planning methodology used for original plans utilizing two coplanar arcs

Patients were treated using plans generated in the Eclipse treatment planning system Version 15.6 (Varian Medical Systems/Siemens Healthineers, Palo Alto, CA). Plans were generated for a Varian TrueBeam treatment machine using the photon optimizer. Plans used a 6‐MV photon energy and utilized between two and four coplanar VMAT beams as shown in Figure 1.

**FIGURE 2:**
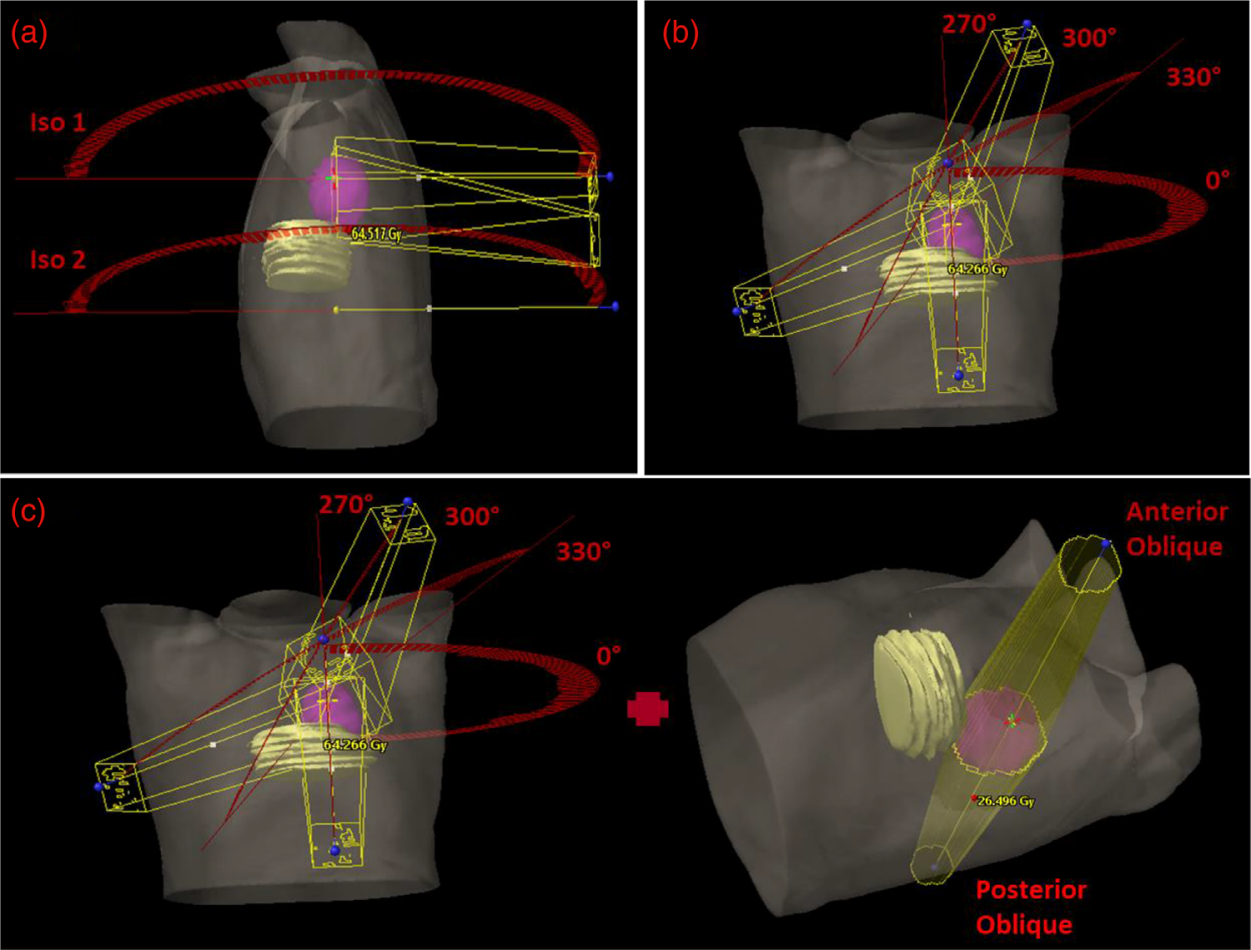
Proposed planning strategies: (a) HEART_MI strategy that utilizes two volume‐modulated arc therapy (VMAT) arcs at two isocenters with ∼10‐cm difference along the Sup‐Inf axis. (b) HEART_NCP strategy that utilizes a traditional VMAT arc at couch 0 along with anterior arcs at up to three noncoplanar delivery angles, and (c) HEART_HYBRID approach that utilizes the HEART_NCP strategy for two thirds of treatments and an opposite‐opposed approach with beams that avoid overlapping the heart for the remaining one third

The plans utilizing the investigational treatment planning strategies were generated by one certified medical dosimetrist with 3‐year planning experience and one board certified medical physicist with 6 years of clinical experience. The planning strategies evaluated were selected based on the ability to minimize geometric overlap of the beam path and the contoured heart structure. Several different strategies were tested. Those presented here showed the most promise at reducing cardiac dose and are deliverable. These techniques were evaluated to determine which would perform best and in what circumstances the others might provide advantages. Plans used for patient treatments were replanned with the same photon energy but incorporating the following planning strategies (see Figure 2) intended to reduce heart dose, while maintaining target coverage and keeping OAR dose within constraints. The first planning strategy was a multi‐isocentric (MI) VMAT approach (HEART_MI). The first isocenter is at a standard location within the tumor, and the second isocenter was chosen up to 10 cm inferiorly or superiorly to the original with the choice of distance among isocenters based on patient anatomy. As demonstrated in Figure 2a, this was done in order to minimize overlap of the beam by directing it above or below the heart depending on the relative position of the lesion and the heart. The distance chosen minimized the geometric overlap of the treatment field with the heart, and a half beam block on the second arc was utilized to further reduce divergence of the beam into the heart. This typically resulted in choosing the largest distance from the initial isocenter possible with the constraint of the 10‐cm limit typically due to the longitudinal extent of the treatment planning CT. Only the two arcs were used in order to maximize the reduction in cardiac dose. At each isocenter, one or two coplanar arcs were utilized for planning.

The second planning strategy was a noncoplanar (NCP) VMAT approach (HEART_NCP) that utilized up to three large couch angles (typically using either 30–60–90‐ or 330–300–270‐degree couch kicks depending on the side of the lesion) in combination with a standard arc at a couch angle of 0 degree (Figure 2b). Small‐angled (typically around 45 degree) gantry arcs over the anterior portion of the patients were used at the large couch angles in order to avoid collisions between the gantry and couch or patient. Just as with the HEART_MI strategy, this strategy attempts to identify advantageous angles for minimizing geometric overlap of the treatment field with the heart, but by rotating the patient instead of shifting the patient longitudinally. The final planning strategy took a mixed approach (HEART_HYBRID) that utilized the HEART_NCP strategy for two thirds (20–22 fx) of the treatment combined with a plan that utilized a pair of opposite‐opposed gantry angles for the remaining one third of patient treatments (10–11 fx) (Figure 2c). The opposite‐opposed gantry angles were selected to minimize overlap of the beams with the heart in order to further push the dose reduction to the heart, and the proportion of each plan that constituted the total treatment was determined in preliminary evaluation in order to maintain an acceptable level of conformality.

In this study, the normal tissue constraints from protocol RTOG 1106–6697^24^ were utilized, and target criterion was 60–66 Gy (2 Gy f*xn*) as requested in dose prescriptions. Typical criteria listed include the percent volume of a structure receiving greater than *x* Gy dose (*Vx*Gy) and the max dose in gray (Gy) to *x* volume of a structure (*Dx*%). Target criteria used in this evaluation included ITV and PTV *D*95% and *D*max, which were parameters listed in the physician's prescription for assessing coverage and homogeneity, as well as *D*98% to assess the near minimum dose to the targets. The full lists of normal tissue constraints used for this study are listed in Table 2. Listed constraints are taken from institutional prescription constraints that are based on RTOG 1106. Additionally, *V*5Gy of the heart was evaluated due to the significance of the correlation between *V*5Gy and cardiac toxicity in several studies (including RTOG 0617). The optimization constraints (including hard and soft constraints) from the original plans were carried over to the new plans. Dose conformity to and fall‐off outside the PTV was also assessed using the conformity index (CI) determined in two ways. We used the RTOG defined as the ratio between the volume covered by the reference isodose and the target volume. For this study, the CI was calculated by evaluating the conformity of the 95% prescription isodose (CI_95_) as in the RTOG convention (CI_95_ = PTV volume/95% prescription isodose),^25^ and also using Paddick's CI formulation that uses the prescription isodose (CI_R_ *_x_*) as the reference isodose and takes into account the amount of overlap of the reference isodose and the target volume (CI_R_ *_x_* = [overlap of PTV and prescription isodose]^2^/[PTV volume × volume of prescription isodose]).^26^ Optimization objectives were kept the same among all treatment strategies except for the heart optimization objectives, which were adjusted in order to achieve the best heart dose while maintaining plan quality. Dosimetric differences between each of the trial planning strategies was compared to the original plan. Dose distributions generated using the various planning strategies were reviewed by a radiation oncologist to assess the acceptability of the plan apart from simple DVH metrics. Statistical significance of the change in dosimetric measurements between each of the trial planning strategies and the original plan was assessed using a paired *t*‐test with a significance level of *p* = 0.05.

## 3 RESULTS

Tables 1 and 2 provide a comparison of the dosimetric values for each of the different planning strategies and for the original plan for targets and OARs, respectively. The percentage difference between the value for the original plan and that of the alternative planning strategies is also listed in the table. Every generated plan met the original target constraints specified in the physicians’ prescriptions. As can be seen in Table 1, there are also no statistically significant differences in PTV coverage between any of the experimental strategies and the original plan aside from a small increase in *D*98 coverage for the HEART_NCP strategy (59.8 vs. 59.0 Gy, *p* = 0.03). For the ITV, however, there were small decreases in *D*95 (61.3 vs. 62.1 Gy [*p* < 0.001]) and *D*98 (60.9 vs. 61.7 Gy [*p* < 0.001]) for the HEART_MI strategy. This is also reflected in that both CI_95_ (1.09 for HEART_MI vs. 1.18, respectively) and CI_R_ *_x_* (0.41 vs. 0.52, respectively) scores are lower compared to the original plan as seen in Table 1, indicating that, although these plans were more conformal with respect to the 95% isodose (less overcoverage), there was a sharper falloff after achieving the prescription constraint, leading to the decrease in *D*98 and lower coverage of the higher prescription isodose. This was largely caused by difficulty in achieving coverage on the side of the target in the cranial–caudal axis opposite the shifted isocenter. By contrast, there were small increases in *D*95 (63.0 vs. 62.1 Gy [*p* = 0.04]) and *D*98 (62.5 vs. 61.7 Gy [*p* = 0.02]) for the HEART_NCP strategy. In this case, the NCP delivery angles included more advantageous treatment directions that made it possible to more readily achieve target coverage goals. As seen in Table 1, this resulted in a more conformal plan in terms of CI_95_, where there was less over‐coverage of the 95% isodose compared to the original plan (1.11 vs. 1.18, respectively), whereas the CI_R_ *_x_* values also reflected better coverage and conformity of the prescription isodose (0.73 vs 0.56). Although the target coverage differences were statistically significant for *D*95 and *D*98, they are generally much less than 1 Gy over the total treatment course (60–66 Gy) and therefore unlikely to be clinically significant.

**TABLE 1:** Comparison of the original target dosimetric values with those of HEART_MI, HEART_NCP, and HEART_HYBRID

		Original plan	HEART_MI		HEART_NCP		HEART_HYBRID	
Targets		Mean (StDev)	Mean (StDev)	Difference	Mean (StDev)	Difference	Mean (StDev)	Difference
PTV	*D*95 (Gy)	60.1 (4.4)	60.0 (3.9)	−0.1 (1.5)	60.9 (4.0)	0.8 (1.3)	60.1 (3.9)	−0.0 (1.4)
PTV	*D*98 (Gy)	59.0 (4.5)	59.0 (3.9)	−0.0 (1.7)	*59.8 (4.0)*	*0.8 (1.4)*	59.0 (3.9)	−0.1 (1.6)
PTV	*D*max (Gy)	67.3 (4.6)	67.6 (4.6)	0.2 (1.7)	*67.6 (4.5)*	*0.3 (1.5)*	67.1 (4.4)	−0.2 (2.3)
PTV	CI_95_	1.18 (0.8)	1.09 (0.1)	−0.9 (0.8)	1.11 (0.11)	−0.07 (0.8)	1.12 (0.13)	−0.06 (0.8)
PTV	CI_R_ *_x_*	0.52 (0.21)	*0.41 (0.19)*	−*0.12 (0.2)*	*0.73 (0.08)*	*0.21 (0.2)*	0.52 (0.16)	0 (0.21)
ITV	*D*95 (Gy)	62.1 (4.3)	*61.3 (4.1)*	−*0.8 (1.1)*	*63.0 (4.0)*	*0.9 (1.9)*	62.0 (4.0)	−0.1 (1.3)
ITV	*D*98 (Gy)	61.7 (4.2)	*60.9 (4.1)*	−*0.8 (1.1)*	*62.5 (4.0)*	*0.8 (1.4)*	61.5 (4.0)	−0.2 (1.4)
ITV	*D*max (Gy)	66.8 (4.6)	66.6 (4.4)	−0.2 (1.7)	66.6 (4.6)	−0.2 (1.1)	66.7 (4.4)	−0.1 (1.9)

*Note*: Mean and standard deviation for each constraint and planning strategy are given as well as the mean and standard deviation of the absolute difference between each novel planning strategy and the original plan values. Statistically significant values are bolded and italicized.

Abbreviation: ITV, internal target volume.

**TABLE 2:** Comparison of the original organ‐at‐risk (OAR) dosimetric values with those of HEART_MI, HEART_NCP, and HEART_HYBRID

				Original plan	HEART_MI		HEART_NCP		HEART_HYBRID	
OARs				Mean (StDev)	Mean (StDev)	Difference	Mean (StDev)	Difference	Mean (StDev)	Difference
Heart	*D*0.03cm^3^ (Gy)	⇐	70	64.2 (6.9)	63.7 (6.8)	−0.5 (3.3)	63.9 (6.6)	−0.3 (3.9)	*62.3 (5.9)*	−1.9 (3.2)
Heart	*V*5Gy (%)			76.5 (19.9)	*54.8 (14.9)*	−*21.6 (11.6)*	*52.0 (11.6)*	−*24.4 (15.1)*	*42.6 (8.3)*	−33.9 (17.6)
Heart	*V*30Gy (%)	⇐	50	13.1 (7.1)	*6.2 (3.9)*	−*6.9 (6.1)*	*5.8 (3.2)*	−*7.3 (6.0)*	*5.0 (2.9)*	−8.1 (5.8)
Heart	*V*50Gy (%)	⇐	25	3.3 (2.8)	*2.3 (2.0)*	−*1.0 (1.7)*	*2.2 (1.9)*	−*1.1 (1.7)*	*2.3 (2.0)*	−1.0 (1.7)
Heart	*D*mean (Gy)	⇐	20	15.4 (4.1)	*9.8 (2.0)*	−*5.6 (3.4)*	*9.2 (1.9)*	−*6.2 (3.4)*	*7.9 (1.5)*	−7.5 (3.4)
Lungs‐ITV	*V*20Gy (%)	⇐	35	24.6 (7.2)	*26.2 (8.0)*	*1.6 (3.0)*	25.9 (7.0)	1.4 (3.5)	*27.9 (9.1)*	*3.4 (6.1)*
Lungs‐ITV	*V*5Gy (%)	⇐	65	62.1 (7.9)	64.0 (12.1)	2.0 (9.2)	64.6 (12.9)	2.5 (10.7)	59.3 (14.8)	−2.8 (13.1)
Lungs‐ITV	*D*mean (Gy)	⇐	20	14.4 (3.1)	14.8 (3.3)	0.4 (1.4)	14.8 (3.2)	0.3 (1.4)	14.5 (3.7)	0.1 (3.3)
Esophagus	*D*0.03cm^3^ (Gy)	⇐	68	52.7 (16.1)	54.8 (14.0)	2.1 (4.6)	54.4 (15.7)	1.7 (4.4)	54.1 (15.0)	1.4 (6.7)
Esophagus	*D*2cm^3^ (Gy)	⇐	63	44.5 (15.8)	45.8 (14.7)	1.3 (5.3)	44.7 (16.1)	0.2 (5.7)	45.4 (16.8)	0.9 (8.6)
Esophagus	*D*mean (Gy)	⇐	34	20.6 (9.8)	20.5 (9.3)	−0.2 (4.6)	19.9 (9.7)	−0.8 (4.6)	19.9 (10.7)	−0.7 (5.7)
Spinal cord	*D*0.03cm^3^ (Gy)	⇐	45	32.3 (10.0)	34.0 (7.3)	1.7 (5.5)	31.4 (7.8)	−0.9 (5.0)	*40.0 (4.9)*	*7.7 (6.9)*

*Note*: Mean and standard deviation for each constraint and planning strategy are given as well as the mean and standard deviation of the absolute difference between each novel planning strategy and the original plan values. Statistically significant values are bolded and italicized.

Abbreviation: ITV, internal target volume.

**FIGURE 3:**
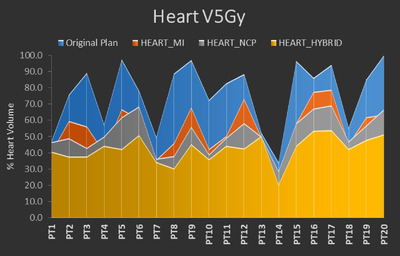
Graph showing the % volume of the heart receiving 5 Gy (*V*5Gy) for the original plan (blue) for each patient overlaid with the corresponding *V*5Gy for the HEART_MI (red), HEART_NCP (gray), and HEART_HYBRID (yellow) strategies

**FIGURE 4:**
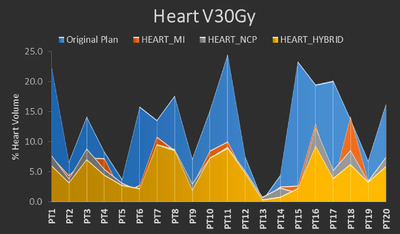
Graph showing the % volume of the heart receiving 30 Gy (*V*30Gy) for the original plan (blue) for each patient overlaid with the corresponding *V*30Gy for the HEART_MI (red), HEART_NCP (gray), and HEART_HYBRID (yellow) strategies

**FIGURE 5:**
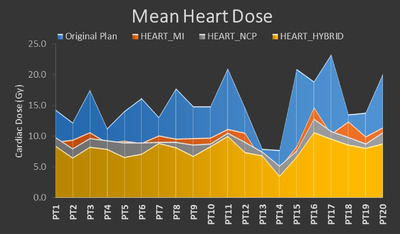
Graph showing the mean heart dose (MHD) for the original plan (blue) for each patient overlaid with the corresponding MHD for the HEART_MI (red), HEART_NCP (gray), and HEART_HYBRID (yellow) strategies

The reduction in cardiac dose is clearly displayed in Table 2. The dosimetric measurements for the heart are listed at the top of Table 2 where there were statistically significant decreases in Heart *V*5, *V*30, *V*50, and mean dose for the HEART_MI (54.8% vs. 76.5% [*p* < 0.001], 6.2% vs. 13.1% [*p* < 0.001], 2.3% vs. 3.3% [*p* = 0.01], and 9.8 vs. 15.4 Gy [*p* < 0.001], respectively), HEART_NCP (52.0% vs. 76.5% [*p* < 0.001], 5.8% vs. 13.1% [*p* < 0.001], 2.2% vs. 3.3% [*p* = 0.02], and 9.2 vs. 15.4 Gy [*p* < 0.001], respectively), and HEART_HYBRID (42.6% vs. 76.5% [*p* < 0.001], 5.0% vs. 13.1% [*p* < 0.001], 2.3% vs. 3.3% [*p* = 0.02], and 7.9 vs. 15.4 Gy [*p* < 0.001], respectively) strategies. Additionally, there was also a statistically significant decrease in Heart max dose (62.3 vs. 64.2 Gy, *p* = 0.02) for the HEART_HYBRID strategy. Particularly of note due to the correlation between these factors and cardiac adverse events, Figures 3, 4, 5 provide a comparison of the reduction in Heart *V*5Gy, *V*30 Gy, and MHD, respectively, for each patient. These were selected due to their importance in correlating with cardiac adverse events in previous studies. In those figures, original plan dosimetric values in blue are overlaid with the values for each of the alternative planning strategies to picture the reduction in value per patient. Using the HEART_MI strategy, MHD was reduced below 10 Gy for 12 of the patients, whereas the number for HEART_NCP was 16 patients and HEART_HYBRID was 18 patients.

For the non‐heart OARs, there were no significant differences in esophagus or lung dose aside from an increase in lung *V*20 for HEART_MI (26.2% vs. 24.6%, *p* = 0.03) and HEART_HYBRID (27.9% vs. 24.6%, *p* = 0.02) that were both a result of an increased overlap of the beam path and lung due to the larger angles used to minimize heart overlap. Similarly, there were no significant differences in the maximum dose to the spinal cord aside from the increase when using the HEART_HYBRID strategy (32.3 vs. 40.0 Gy, *p* < 0.001), which was a result of the contribution of the portion of the treatment that utilized an opposite‐opposed beam arrangement that often overlapped the spinal cord to find an optimal angle for avoiding the heart. Overall, average monitor units (MUs) for the original plan was 513.6 ± 117.6 MU, whereas the average was 705.2 ± 89.7, 623.6 ± 102.6, and 623.6 ± 102.6 MU (NCP portion)/291.0 ± 53.4 MU (opposite‐opposed portion) for the HEART_MI, HEART_NCP, and HEART_HYBRID strategies, respectively.

## 4 DISCUSSION

In this study, three planning strategies were retrospectively implemented in order to assess their feasibility at reducing dose to the heart and ultimately cardiac toxicity. Each of the planning strategies was able to achieve reduced heart dose while maintaining target coverage and meeting listed OAR constraints, though with trade‐offs that were unique to each of them. The MI approach (HEART_MI) exhibited the smallest dose reduction but was able to reduce the mean dose below 10 Gy for over half of the patients. Due to the geometry of the approach, there was a small drop in target coverage typically at the superior end of the lesion (opposite the shifted isocenter), and there was a greater amount of overlap of the beam with the lung, and therefore, a higher lung *V*20, though within the listed constraints. Overall, the HEART_NCP plan showed the best conformity even relative to the original plans, which could be attributed to providing advantageous treatment angles to improve conformity. As expected, this did lead to an increase in the low dose spread (*V*5Gy) in the anterior region of the patient, which can be observed in the larger lung *V*5 when using the HEART_NCP strategy (2.5% increase over original plans). For the HEART_MI plans, the conformity was similar to the original plan overall. However, there was a bit of an increase in the spread of dose superior and inferior to the target due to the angle of the off‐isocenter arc, partially contributing to lower CI_R_ *_x_* scores for this patient set. In HEART_HYBRID plans, the opposite‐opposed portion of the plan led to a stretching of the dose along the angle of those beams and provided the greatest challenge in terms of clinical acceptability. The strategy that showed the greatest amount of cardiac dose reduction was the mixed plan (HEART_HYBRID), which showed significant decrease in cardiac dose for every metric tested as well as reducing the MHD below 10 Gy for all but two patients. However, particularly because of the contribution of the portion of the treatment that used an opposite‐opposed beam arrangement, this led to a higher *V*20 and spinal cord dose than in the original plans. This was due to the necessity of choosing angles that minimized overlap with the heart which usually led to beam paths that passed through the contralateral lung and directly through the spinal cord.

The planning strategy that seemed to exhibit the optimal reduction in dose to the heart without leading to significant differences for other OARs was the NCP strategy (HEART_NCP). However, this planning strategy required a greater amount of complexity relative to alternative methods both in terms of treatment planning and treatment setup and delivery. This was reflected in the average increase of about 100 MU (or 20%) relative to the patients’ original plans. Additionally, the care taken to avoid collisions of the gantry plus the increased number of couch rotations during treatment would put a greater strain on departmental resources. As mentioned earlier, MHD was highly dependent on volume and length as well as distance of the lesion from the target. Overall, it appeared that HEART_NCP strategy provided the best conformity as reflected in the CI_95_ and CI_R_ *_x_* scores while also providing a significant level of cardiac dose reduction. However, for those lesions closest to the heart, extending along the length of the heart, and where there is room for compromise on the plan conformity, the HEART_HYBRID strategy can provide a level of cardiac dose sparing that the other two strategies struggle with. The HEART_MI strategy performed best for smaller lesions where there was a separation longitudinally between the lesion and the heart as in Patient 17.

Not every patient may benefit from these types of nonstandard strategies to minimize cardiac dose. Patients with lesions who only have a small portion may see only minimal dosimetric improvement using these strategies. In this study, Patient 13 showed the smallest dose reduction to the heart with MHD only decreasing in the range from 0.4 Gy using the HEART_NCP strategy to 1.8 Gy using the HEART_MI approach. In that case, the bulk of the lesion was located superior to the heart (∼60%), and the high MHD was driven by that part of the PTV that directly overlapped or abutted the heart and so had minimal potential for improvement without sacrificing target coverage. In this study, patients who received an MHD greater than 10 Gy were selected based on several studies that indicated that an MHD was significantly correlated with an increase in the level of cardiac events,^15^, ^17^, ^18^ and that 10 Gy could be used as a threshold above which, there was a higher risk of cardiac adverse events.^15^ However, several clinical prognostic factors could be utilized to identify patients who may benefit from a more aggressive approach for reducing cardiac dose prior to planning. As mentioned previously, a significant correlation between the presence of preexisting heart conditions and an increase in cardiac adverse events has occurred in several studies.^15^, ^17^, ^18^, ^19^ Patients who exceeded this threshold tended to have larger PTVs with a mean volume greater than 400 cm^3^ (mean: 415.3 cm^3^ [range: 115.83–1184.85 cm^3^]) with mediastinal invasion in the anterior and middle regions of the inferior mediastinum. For our patient set, although no patients with PTV volumes less than 100 cm^3^ exceeded the threshold, patients with PTV volumes between 100 and 200 cm^3^ exceeded the threshold only 23.2% of the time, and 51.8% of patients with PTV volumes greater than 300 cm^3^ exceeded the threshold. PTV volume and location were the two biggest factors that led to excessive cardiac dose for this set of patients, and these two factors could be used as a guide to determine which patients are at highest risk of having excessive cardiac dose. Therefore, the patients who would be expected to have the most benefit are those patients with a higher risk profile due to a combination of clinical factors such as preexisting cardiac disease and dosimetric factors such as the likelihood of a high cardiac dose as a result of size and location of tumor.

Although this paper focuses on the potential benefit that could be derived from using tools that exist within the current treatment planning framework, there exists research into further methods to reduce dose using artificial intelligence–based strategies that have shown promise for finding the optimal solution for mitigating OAR doses. Multiple methods have been proposed for utilizing machine learning to improve dosimetric results in radiotherapy through dose mimicking in head and neck patients,^27^ generating optimal fluence maps that can be imported into the treatment planning system for dose calculation for prostate patients,^28^ and relevantly, for reducing heart dose in lung cancer patients using a machine‐learning approach for assessing trade‐offs between lung and cardiac toxicity during planning.^29^ In this last study, nearly 40% of patients had predicted plans that exhibited mean dose reductions of >4 Gy in MHD. This is a promising area of research, and there exists a great deal of potential in utilizing a machine‐learning guided method to further reduce cardiac dose and ultimately toxicity in this population of patients.

## 5 CONCLUSION

Three planning strategies were introduced that were able to reduce the cardiac dose, while maintaining target coverage compared to the conventional planning strategy and to maintain OAR doses within acceptable levels. The strategy that provided the best compromise between plan quality and cardiac dose reduction utilized large NCP anterior arcs and produced MHD reductions of 37.6% ± 12.9% (6.2 ± 3.4 Gy) relative to original plans. This strategy could be implemented for a set of patients who have been identified as higher risk for cardiac adverse events based on clinical or other prognostic factors, which has the potential to reduce adverse cardiac events and improve the QOL for this subset of patients.

## AUTHOR CONTRIBUTION

Joshua P. Kim was involved in the concept, plan generation, analysis, and writing off the manuscript. Jake Dewalt was involved in the plan generation and writing of the manuscript. Indrin J. Chetty was involved in the conception, analysis, and editing of the manuscript. Aharon Feldman, Khaled Adil, and Benjamin Movsas were involved in the contribution of patients, feedback, and editing of the manuscript.

## CONFLICTS OF INTEREST

HFHS holds research agreements with Philips Healthcare and Varian Medical Systems.

## FUNDING INFORMATION

This work was supported by an Internal Mentored Grant.
